# Dietary Tryptophan Induces Opposite Health-Related Responses in the Senegalese Sole (*Solea senegalensis*) Reared at Low or High Stocking Densities With Implications in Disease Resistance

**DOI:** 10.3389/fphys.2019.00508

**Published:** 2019-05-01

**Authors:** Rita Azeredo, Marina Machado, Juan A. Martos-Sitcha, Gonzalo Martínez-Rodríguez, Joana Moura, Helena Peres, Aires Oliva-Teles, António Afonso, Juan M. Mancera, Benjamín Costas

**Affiliations:** ^1^Interdisciplinary Centre of Marine and Environmental Research, University of Porto, Matosinhos, Portugal; ^2^Institute of Biomedical Sciences Abel Salazar, University of Porto, Porto, Portugal; ^3^Department of Biology, Faculty of Marine and Environmental Sciences, Instituto Universitario de Investigación Marina, University of Cádiz, Cádiz, Spain; ^4^Department of Marine Biology and Aquaculture, Institute of Marine Sciences of Andalusia, Spanish National Research Council, Cádiz, Spain; ^5^Department of Biology, Faculty of Sciences, University of Porto, Porto, Portugal

**Keywords:** amino acid, neuro-endocrine response, functional feed, cortisol, crowding stress

## Abstract

High rearing densities are typical conditions of both inland and onshore intensive aquaculture units. Despite obvious drawbacks, this strategy is nonetheless used to increase production profits. Such conditions inflict stress on fish, reducing their ability to cope with disease, bringing producers to adopt therapeutic strategies. In an attempt to overcome deleterious effects of chronic stress, Senegalese sole, *Solea senegalensis*, held at low (LD) or high density (HD) were fed tryptophan-supplemented diets with final tryptophan content at two (TRP2) or four times (TRP4) the requirement level, as well as a control and non-supplemented diet (CTRL) for 38 days. Fish were sampled at the end of the feeding trial for evaluation of their immune status, and mortalities were recorded following intra-peritoneal infection with *Photobacterium damselae* subsp. *piscicida*. Blood was collected for analysis of the hematological profile and innate immune parameters in plasma. Pituitary and hypothalamus were sampled for the assessment of neuro-endocrine-related gene expression. During the feeding trial, fish fed TRP4 and held at LD conditions presented higher mortalities, whereas fish kept at HD seemed to benefit from this dietary treatment, as disease resistance increased over that of CTRL-fed fish. In accordance, cortisol level tended to be higher in fish fed both supplemented diets at LD compared to fish fed CTRL, but was lower in fish fed TRP4 than in those fed TRP2 under HD condition. Together with lower mRNA levels of *proopiomelanocortin* observed with both supplementation levels, these results suggest that higher levels of tryptophan might counteract stress-induced cortisol production, thereby rendering fish better prepared to cope with disease. Data regarding sole immune status showed no clear effects of tryptophan on leucocyte numbers, but TRP4-fed fish displayed inhibited alternative complement activity (ACH50) when held at LD, as opposed to their HD counterparts whose ACH50 was higher than that of CTRL-fed fish. In conclusion, while dietary tryptophan supplementation might have harmful effects in control fish, it might prove to be a promising strategy to overcome chronic stress-induced disease susceptibility in farmed Senegalese sole.

## Introduction

Every physiological response (e.g., metabolism, mineral balance, reproduction, growth, and immune function) is at least partly regulated by neuroendocrine mechanisms, which, in teleosts, are orchestrated by the hypothalamus-pituitary-interrenal (HPI) axis. Neuroendocrine signals are released upon stimulation of HPI axis and responses are highly different within each context. In fish, the stress response, triggered by both internal and external signals, consists of the production and secretion of different molecules, such as the corticotropin-releasing hormone (Crh) and Crh-binding protein (Crhbp) in the hypothalamus, adrenocorticotropic hormone (ACTH) in the pituitary, and cortisol in the head-kidney interrenal tissue (Bonga, 1997; Flik et al., 2006). The latter has been generally recognized as the most important stress indicator in fish (Tort, 2011) and, amongst other intermediates, ACTH is the ultimate cortisol secretion-inducer. ACTH is a polypeptide hormone encoded in the medium region of the proopiomelanocortin gene (*pomc*) which post-translational processing yields not only ACTH, but γ, α and β-melanocyte-stimulating hormone (MSH) and β-endorphin (Takahashi et al., 2013; Navarro et al., 2016). In most teleost species, given the earlier genome duplication, two forms of proopiomelanocortins are present, named *pomca* and *pomcb*. In Senegalese sole (*Solea senegalensis*) the two isoforms have been shown to depict subfunctionalization and therefore are expected to be differently modulated by stress (Wunderink et al., 2012). HPI axis activity is also indirectly modulated by serotonin, which receptors activity seem to both stimulate and inhibit ACTH secretion in fish, hence, cortisol release (Höglund et al., 2002).

Being a precursor of the monoamine serotonin, tryptophan availability dictates serotonin production and so can indirectly (through adrenocorticotropic hormone action) affect stress response in fish, as already reviewed in several fish species (Hoseini et al., 2017). In fact, an increased tryptophan availability in the diet was already reported to affect endocrine and behavioral responses to stress in fish (Winberg et al., 2001; Lepage et al., 2002; Höglund et al., 2007), including the Senegalese sole (Costas et al., 2012). The later study also highlighted the importance of feeding time, a fact already reported previously for the rainbow trout (*Oncorhynchus mykiss*) (Lepage et al., 2003) and also reviewed by Hoseini et al. (2017). Nevertheless, the nature of stress factors that effectively activates an HPI response is wide-ranging and it eventually plays a role in dictating response development (Martos-Sitcha et al., 2014). While dietary supplementation of tryptophan lowered cortisol levels in acute crowding stressed rainbow trout (Lepage et al., 2002), this effect seems to not so clear during longer periods of time (Hoseini et al., 2017). Having this in mind, it remains to be elucidated whether a long-term tryptophan feeding presents a clear role on the modulation of the HPI axis response under chronic stressful conditions in fish.

The importance of neuroendocrine-immune interactions is not unidirectional. Not only immune stimulation has the ability to trigger a neuroendocrine response. Indeed, the presence of nerve fibers innervating teleosts head-kidney, and cortisol receptors in leucocytes, show the importance of neuroendocrine mechanisms in regulating other physiological processes such as the immune response (Tort, 2011). Cortisol well-known immunosuppressive effects in fish (Saeij et al., 2003; Varsamos et al., 2006), and the immune-mediated cortisol release (Haukenes and Barton, 2004) clearly illustrate the abovementioned relationship, particularly when stressful situations last through time and turn into chronic stress conditions.

Chronic stress imposed by high rearing densities in farmed fish and daily husbandry routines may impair immune defenses and thereby increase fish susceptibility to disease (Tort, 2011). It is therefore of critical importance to find and adopt strategies to minimize the impact of such aquaculture-related practices on fish health. A significant effort is being made to find compounds that display immune-enhancing properties, such as pro- and prebiotics, vitamins, minerals, fatty acids, etc. (Manning and Gibson, 2004; Li et al., 2009; Nayak, 2010). Given their versatility in terms of biological roles they play, amino acids dietary supplementation is becoming an important research topic in fish immunonutrition. Within amino acids, the potential benefit of tryptophan in the diet was investigated in different fish species (reviewed by Hoseini et al., 2017; Höglund et al., 2019), although the results are not yet conclusive. Therefore, this study was conceived to evaluate the potential benefits of tryptophan dietary surplus at different levels on Senegalese sole neuroendocrine and metabolic response in a factorial design (3 × 2) in fish held at low or high stocking densities as a chronic stress source. Furthermore, the present work aims to unravel possible effects of dietary tryptophan supplement on Senegalese sole immune condition and disease resistance to a bacterial infection.

## Materials and Methods

### Experimental Diets

A practical diet was formulated to be isonitrogenous and isolipidic (45% crude protein and 16% crude fat) and to meet the estimated Senegalese sole amino acids profile, and served as control (CTRL). Two other diets were formulated similar to CTRL and supplemented with 0.86 and 1.72% tryptophan (diets TRP2 and TRP4, respectively). Lowest supplementation level was selected based on preliminary studies in which cortisol-mediated immunosuppression was reduced (Costas et al., 2012, 2013b). Lepage et al. (2002) on rainbow trout demonstrated tryptophan role in counteracting cortisol elevation when dietary inclusion levels were as high as eightfold the tryptophan requirement level. This study served as a reference to the use of a higher (4×) tryptophan supplementation level. The essential amino acids profile from the control diet was formulated considering the requirement previously determined for Senegalese sole juveniles (Silva et al., 2010). In the absence of specific data on tryptophan requirement of Senegalese sole, requirement data for other species were applied (Kaushik, 1998).

Detailed information on diet composition and proximate analysis is given in Table 1. All ingredients were ground, mixed together and dry-pelleted in a laboratory pellet mill (CPM, California Pellet Mill, Crawfordsville, IN, United States). Proximate analysis of the diets was performed according to the Association of Official Analytical Chemists methods (AOAC, 2000) and amino acids analysis was carried out according to Machado et al. (2015) and tryptophan was measured by a spectrophotometric method as described by De Vries et al. (1980). Amino acids composition of the diets is presented in Table 2.

**Table 1:** Ingredients and proximate composition of the experimental diets.

	Experimental diets		
Ingredients (% dry weight)	CTRL	TRP2	TRP4
Fish meal^a^	25	25	25
CPSP^b^	5.0	5.0	5.0
Corn gluten^c^	20	20	20
Soybean meal^d^	14.4	12.9	11.3
Wheat meal^e^	18.2	18.8	19.5
Cod liver oil	11.7	11.7	11.7
Vitamin mix^f^	1.0	1.0	1.0
Mineral mix^g^	1.0	1.0	1.0
Choline chloride (50%)	0.50	0.50	0.50
Binder (Aquacube)^h^	1.0	1.0	1.0
Agar	1.0	1.0	1.0
Tryptophan		0.86	1.72
**Proximate analysis (% dry weight)**			
Dry matter (%)	89.3	89.2	89.6
Crude protein	47.1	47.1	47.0
Crude fat	16.4	16.3	16.4
Ash	10.2	10.9	10.1

*^a^Steam dried LT fish meal, Pesquera Diamante, Perú (CP: 70.7% DM; CF: 9.1% DM). ^b^Soluble fish protein concentrate; Sopropêche, France (CP: 80.4% DM; CF: 19.7% DM). ^c^Sorgal, Portugal (CP: 68.3% DM; CF: 2.9% DM). ^d^Sorgal, Portugal (CP: 53.5% DM; CF: 1.0% DM). ^e^Sorgal, Portugal (CP: 14.1% DM; CF: 2.2% DM). ^f^Vitamins (mg kg^−1^ diet): retinol acetate, 18000 (IU kg^−1^ diet); cholecalciferol, 2000 (IU kg^−1^ diet); alpha tocopherol acetate, 35; sodium menadione bisulfate, 10; thiamin-HCl, 15; riboflavin, 25; calcium pantothenate, 50; nicotinic acid, 200; pyridoxine HCl, 5; folic acid 10; cyanocobalamin, 0.02; biotin, 1.5; ascorbic acid, 50; inositol, 400. ^g^Minerals (mg kg^−1^ diet): cobalt sulfate, 1.91; copper sulfate, 19.6; iron sulfate, 200; sodium fluoride, 2.21; potassium iodide, 0.78; magnesium oxide, 830; manganese oxide, 26; sodium selenite, 0.66; zinc oxide, 37.5; dibasic calcium phosphate, 5.93 (g kg^−1^ diet); potassium chloride, 1.15 (g kg^−1^ diet); sodium chloride, 0.40 (g kg^−1^ diet). ^h^Agil, England (guar gum, polymethyl carbamide, manioc starch blend, hydrate calcium sulfate).*

**Table 2:** Determined amino acid composition (g 16 g^−1^ N) of the experimental diets.

	Experimental diets		
Amino acid analysis (g 16 g^−1^ protein)	CTRL	TRP2	TRP4
Lysine	5.54	5.57	5.41
Arginine	5.22	5.14	5.17
Histidine	2.99	2.91	2.86
Isoleucine	4.67	4.54	4.47
Leucine	7.62	7.50	7.37
Valine	7.11	6.88	6.89
Methionine	2.08	2.06	2.08
Phenylalanine	3.66	3.59	3.55
Threonine	6.07	6.04	6.00
Tyrosine	2.57	2.62	2.61
Aspartic Acid	8.18	8.15	8.22
Glutamic acid	16.4	16.1	16.1
Serine	5.62	5.64	5.27
Glycine	6.64	6.49	6.25
Alanine	7.54	7.36	7.13
Proline	7.12	7.14	7.09
Tryptophan	0.97	2.28	4.36

### Fish and Experimental Design

This study was carried out at the Interdisciplinary Centre of Marine and Environmental Research facilities, Porto, Portugal. Healthy, non-vaccinated Senegalese sole (45.3 ± 0.3 g) juveniles were obtained from a Portuguese commercial fish farm with no history of photobacteriosis. After 2 weeks of acclimatization, fish were weighed, measured and distributed to 18 flat-bottomed, shaded tanks (0.05 m^2^; 6.5 L) of two separate seawater recirculation systems [temperature: 20 ± 1°C; salinity: 24 ppt; natural light-dark cycle (June, 2013, 41°09′07.0″N 8°36′56.1″W)]. Fish were distributed to establish two different stocking densities, one per system: in one of the systems, each of 9 tanks held 14 fish (12.5 kg m^−2^, LD group) and was considered the control group, while in the other system, each of 9 tanks held 34 fish (31 kg m^−2^, HD group) and fish were regarded as chronically stressed. It is noteworthy to mention that LD cannot be considered as unstressed animals but fish undergoing a lower degree of stress. Density levels were chosen in accordance to that currently used in super-intensive production systems for Senegalese sole. Dietary treatments were randomly assigned to triplicate tanks of each density group. Fish were fed twice a day (9 am and 4 pm) until apparent satiety for 38 days, and water parameters, including nitrogenous compounds, were monitored daily.

Fish were fasted 24 h prior to sampling to avoid any influence of feeding on cortisol and glucose levels (Arends et al., 1999). At day 39, 4 fish per tank (12 fish per dietary treatment, and per density) were killed by immersion in 2-phenoxyethanol (1 mL L^−1^; Sigma). Blood was collected from the caudal vessel with heparinized syringes and processed for hematological analyses as described below. The remaining blood was centrifuged for 10 min at 10,000 × *g* and 4°C and then stored at −80°C until further analyses. Pituitary and hypothalamus were also collected and kept in a 1/10-relation w/v of RNAlater^®^ stabilization solution (Ambion Inc., Austin, TX, United States) at 4°C for 24 h and then stored at −80°C until assayed. For gene expression purposes, two fish per tank were used (*n* = 6).

The experiments were approved by the Animal Welfare Committee of the Interdisciplinary Centre of Marine and Environmental Research and carried out in a registered installation (N16091.UDER). Experiments were performed by trained scientists in full compliance with national rules and following the European Directive 2010/63/EU of the European Parliament and the European Union Council on the protection of animals used for scientific purposes.

### Bacteria Inoculum Preparation

*Photobacterium damselae* subsp. *piscicida* (*Phdp*) strain PC566.1 was isolated from Senegalese sole and kindly provided by Professor Alicia E. Toranzo (Departamento de Microbiología y Parasitología, Facultad de Biología, University of Santiago de Compostela, Spain). Stocked bacteria were cultured for 48 h at 22°C on tryptic soy agar supplemented with NaCl at 1% (TSA-1) and then inoculated into tryptic soy broth similarly supplemented with NaCl at 1% (TSB-1) (both media from Difco Laboratories) and cultured overnight at the same temperature, with continuous shaking (100 rpm, Rotator DSR2100V). Exponentially growing bacteria were collected by centrifugation at 3,500 × *g* for 30 min, resuspended in sterile phosphate-buffered saline (PBS) and adjusted to a LD_50_ [5 × 10^3^ colony forming units (CFU) mL^−1^] by reading absorbance against a growth curve, according to Costas et al. (2013a). Final bacterial concentration was confirmed by plating serial dilutions onto TSA-1 plates and counting the number of CFU after incubation at 22°C for 48 h.

### Bacterial Challenge

At the end of the feeding trial, 20 fish per treatment (i.e., density and diet) were intraperitoneally injected with either 100 μL *Phdp* (5 × 10^3^ CFU mL^−1^) or 100 μL PBS and redistributed to duplicate tanks (five fish per tank) of four independent seawater recirculation systems under the same conditions described above. Feeding protocol and daily maintenance were kept as for the pre-challenge trial except for water temperature that was increased to 22°C to simulate a possible scenario during natural outbreaks (Arijo et al., 2005). Fish mortality was recorded for 3 weeks. Moribund or dead fish were removed, weighed, and sampled for bacteria isolation by plating head-kidney samples onto TSA-1 plates.

### Analytical Procedures With Blood and Peripheral Leucocytes

An aliquot of gently homogenized blood was used to perform total white blood cells (WBC) and red blood cells (RBC) counts, hematocrit (Ht), and hemoglobin (Hb; SPINREACT kit, ref. 1001230, Spain) determinations. Mean corpuscular volume (MCV), mean corpuscular hemoglobin (MCH) and mean corpuscular hemoglobin concentration (MCHC) were also calculated as follows:

– MCV (mm^3^) = (Ht/RBC) × 10
– MCH (pg cell^−1^) = (Hb/RBC) × 10
– MCHC (g 100 mL^−1^) = (Hb/Ht) × 100

Blood smears were also prepared from fresh homogenized blood, air dried and stained with Wright’s stain (Hemacolor; Merck) after fixation with formaldehyde-ethanol (10% of 37% formaldehyde in absolute ethanol). The slides were examined (1000×) and at least 200 leucocytes were counted per smear and classified as thrombocytes, lymphocytes, monocytes, and neutrophils. Peroxidase activity was carried out as described by Afonso et al. (1998) in order to facilitate identification of neutrophils. Absolute value (×10^4^ mL^−1^) of each cell type was subsequently calculated.

### Plasma Cortisol

Plasma cortisol levels were measured using enzyme-linked immunosorbent assays (ELISAs) performed in microtitre plates (MaxiSorp^TM^, Nunc, Roskilde, Denmark), as previously described by Martos-Sitcha et al. (2014) for other teleost species. Steroids were extracted from 5 μL plasma in 100 μL RB [10% v/v potassium phosphate buffer (PPB) 1 M, 0.01% w/v NaN_3_, 2.34% w/v NaCl, 0.037% w/v EDTA, and 0.1% w/v bovine serum albumin (BSA)] and 1.2 mL methanol (Panreac), which was then allowed to evaporate over 48–72 h at 37°C. Cortisol ELISA standard (Cat. #10005273), goat anti-mouse IgG monoclonal antibody (Cat. #400002), specific cortisol express ELISA monoclonal antibody (Cat. #400372), and specific cortisol express AChE tracer (Cat. #400370) were obtained from Cayman Chemical Company (MI, United States). Standards and extracted plasma samples were analyzed in duplicate. The standard curve was constructed through 1:1 serial dilutions from 2.5 ng mL^−1^ to 9.77 pg mL^−1^ (*R*^2^ = 0.992). The lower limit of detection (95.85% of binding, ED98.75) was 14.6 pg mL^−1^. The percentage of recovery was 95%. Inter- and intra-assay coefficients of variation (calculated from sample duplicates) were 3.67 ± 1.18% and 2.89 ± 0.63%, respectively. Cross-reactivity of the specific antibody toward intermediate steroid synthesis or metabolism products was determined by the supplier (Cayman Chemical Company, MI, United States).

### Plasma Metabolites

Plasma glucose, lactate and triglycerides were assessed using commercially available Spinreact kits (Glucose HK Ref. 1001200; Lactate Ref. 1001330; Triglycerides Ref. 1001311; Sant Esteve d’en Bas, Girona, Spain), adapted for 96-well microplates. All assays were carried out on a microplate reader (BioTek Instruments, Winooski, VT, United States) using the KCjunior Data Analysis Software for Microsoft Windows XP.

### Innate Humoral Parameters

Plasma bactericidal activity was measured according to Graham et al. (1988) with some modifications (Machado et al., 2015). Total bactericidal activity is expressed as percentage, calculated from the difference between the dissolved formazan in samples and the one formed in the positive controls (100%). The bactericidal activity was calculated as the percentage of non-viable bacteria.

Alternative complement pathway activity (ACH50) was evaluated as described by Sunyer and Tort (1995). The ACH50 units were defined as the concentration of plasma inducing 50% hemolysis of rabbit red blood cells.

A turbidimetric assay was used to evaluate lysozyme activity following the method as described by Costas et al. (2011).

Total peroxidase activity in plasma was measured following the procedure described by Quade and Roth (1997). Peroxidase activity was determined by defining one unit of peroxidase as that which produces an absorbance change of 1 OD (units mL^−1^ of plasma).

### Gene Expression Analysis

Total RNA was isolated from individual pituitaries using NucleoSpin^®^RNA XS kit (Macherey-Nagel), and from hypothalamus using NucleoSpin^®^RNA II kit (Macherey-Nagel). The on-column RNase-free DNase digestion was used for gDNA elimination. The manufacturer’s instructions were followed in this procedure. Additionally, the amount of RNA was fluorimetrically measured with the Qubit^®^ 2.0 Fluorometer (Invitrogen^TM^, Life Technologies) and its quality was determined in a 2100 Bioanalyzer using the RNA 6000 Nano Kit (Agilent Technologies). Only samples with a RNA Integrity Number higher than 8.5, which is indicative of clean and intact RNA, were used for real-time PCR (qPCR).

Primer oligonucleotide sequences and final concentrations used in the qPCR reactions are shown in Table 3 and were as described by Wunderink et al. (2011, 2012), except for *gr1* and *gr2*, which were designed with NCBI Primer Blast Tool and the oligo analyzer tool of IDT^®^. The efficiency of *gr1* and *gr2* primer pairs was analyzed in serial fivefold dilutions of cDNA by calculating the slope of the regression line of the cycle thresholds versus the relative concentration of cDNA. Total RNA was used to synthesize the first strand cDNA by reverse transcription (RT) reaction using qSCRIPT^TM^ cDNA Synthesis Kit (Quanta BioSciences). The qPCR was carried out with Fluorescent Quantitative Detection System (Eppendorf Mastercycler ep realplex 2 S). Each reaction mixture (10 μL) contained 0.5 μL each specific forward and reverse primer, and 5 μL PerfeCTa SYBR^®^ Green FastMix^TM^ (Quanta Biosciences). Reactions were conducted in semi-skirted twin-tec 96-well real-time PCR plates (Eppendorf) covered with adhesive Masterclear real-time PCR Film (Eppendorf). The thermocycling procedures were as previously described by Wunderink et al. (2011, 2012). Melting curves were used to ensure that only a single PCR product was amplified and to verify the absence of primer-dimer artifacts. Each sample was run in triplicate. The results were normalized to β-actin levels due to its low variability (less than 0.10 C_T_ in each tissue) under our experimental conditions. Relative gene quantification was performed using the ΔΔC_T_ method (Livak and Schmittgen, 2001).

**Table 3:** Specifications of real-time PCR assays including forward (F) and reverse (R) primers, GenBank ID (NCBI), efficiencies (Eff) of PCR reactions, annealing temperature (Ta), and length of amplicon.

Gene	Acronym	GenBank ID	Eff (%)^1^	Ta (°C)	Amplicon length (bp)	Primer sequence (5′–3′)
Corticotropin releasing hormone	*crh*	FR745427.1	98.1	60	135	F: CCTGACCTTCCACCTGCTAC R: GAGATCTTTGGCGGAGTGAA
Corticotropin releasing hormone-binding protein	*crhbp*	FR745428.1	101.3	60	118	F: GGCAATGGCATAGACACCTC R: CACTGGACACCAGCCTCAC
Glucocorticoid receptor 1	*gr1*	AB614369.1	105.6	60	154	F: CATGACGACCCTGAACCGAT R: CCAGCCCAGACTGAAAGACA
Glucocorticoid receptor 2	*gr2*	AB614370.1	100.0	60	150	F: ACCATGCTGTCTGTGCTCAA R: AGACTTGGCCCACTTGACTG
Proopiomelanocortin A	*pomca*	FR851915.1	102.2	60	127	F: AAGGCAAAGAGGCGTTGTAT R: TTCTTGAACAGCGTGAGCAG
Proopiomelanocortin B	*pomcb*	FR851916.1	103.9	60	110	F: GTCGAGCAACACAAGTTCCA R: GTCAGCTCGTCGTAGCGTTT
β-actin	*actb*	DQ485686.1	99.6	60	108	F: TCTTCCAGCCATCCTTCCTCG R: TGTTGGCATACAGGTCCTTACGG

*^1^Efficiency of PCR reactions (represented in percentage) were calculated from serial dilutions of tissue RT reactions in the validation procedure.*

### Statistical Analysis

Statistical analyses were performed with STATISTICA (StatSoft, Inc., 2013, version 12) for WINDOWS. Results are expressed as means ± SD. Data were analyzed for normality and homogeneity of variance and, when necessary, outliers were removed using the STATISTICA tool for outliers and extremes removal. Data were analyzed by Multifactorial ANOVA with dietary treatment and rearing density as variables. Whenever significant differences were found among groups, a multiple-comparisons Tukey HSD test was performed to identify significantly different groups. For every test, the level of significance chosen was *p* ≤ 0.05.

## Results

### Diets and Growth Performance

Experimental diets were well accepted and survival was 100% for all treatments at the end of the experimental period. FBW, SGR, VFI, K, and HSI were not affected by diet composition. K was also not affected by rearing density, whereas FBW, SGR, and HSI were higher in fish reared at LD in contrast to the increased VFI observed in HD fish (Table 4).

**Table 4:** Initial (IBW) and final (FBW) body weight, specific growth rate (SGR), voluntary feed intake (VFI), condition factor (K), and hepatosomatic index (HSI) in Senegalese sole after 38 days held at different treatments.

Parameters	Treatments					
	LD			HD		
	CTRL	TRP2	TRP4	CTRL	TRP2	TRP4
IBW (g)	45.24 ± 0.08	44.98 ± 0.37	45.07 ± 0.47	45.47 ± 0.10	45.49 ± 0.06	45.50 ± 0.21
FBW (g)	51.78 ± 0.78	50.86 ± 0.85	51.47 ± 0.36	48.60 ± 0.15	48.89 ± 0.80	48.67 ± 0.48
SGR (% day^−1^)	0.36 ± 0.05	0.36 ± 0.06	0.39 ± 0.03	0.20 ± 0.01	0.21 ± 0.04	0.20 ± 0.02
VFI	0.19 ± 0.01	0.19 ± 0.01	0.10 ± 0.01	0.30 ± 0.01	0.29 ± 0.01	0.30 ± 0.01
K (g cm^−3^)	1.21 ± 0.01	1.21 ± 0.01	1.21 ± 0.07	1.20 ± 0.06	1.21 ± 0.04	1.21 ± 0.03
HSI (%)	1.31 ± 0.17	1.33 ± 0.32	1.46 ± 0.43	1.09 ± 0.21	1.21 ± 0.32	1.04 ± 0.15

Parameters	Density	Diet	Density × diet	LD	HD
FBW	<0.001	ns	ns	B	A
SGR	<0.001	ns	ns	B	A
VFI	<0.001	ns	ns	A	B
K	ns	ns	ns		
HSI	<0.001	ns	ns	B	A

*Values are expressed as means ± SD (n = 3; except for HSI n = 12). A and B represent significant differences between density treatments regardless of dietary treatment, whereas ns means no significant differences. Two-way ANOVA; Tukey post hoc test; p ≤ 0.05. Voluntary feed intake (VFI) = Crude feed intake/average body weight [(initial body weight+final body weight)/2]/days.*

### Cumulative Mortality in Challenged Fish

**FIGURE 1:**
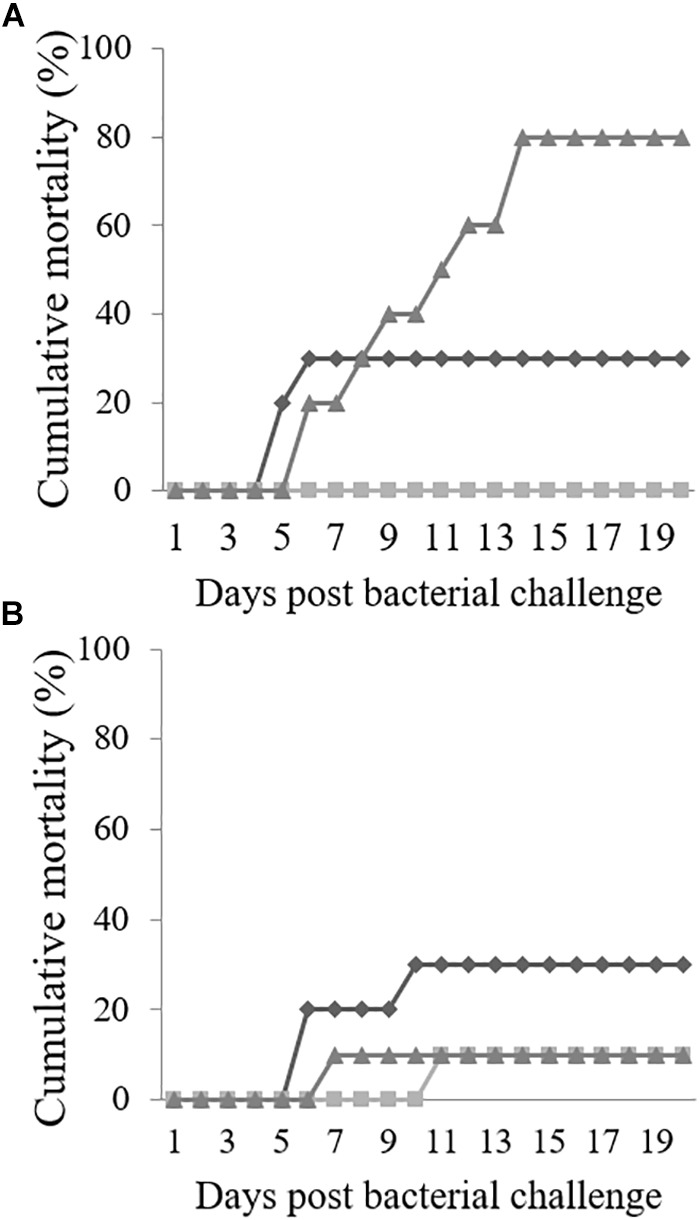
Cumulative mortality (%) of Senegalese sole reared at low **(A)** or high **(B)** stocking densities, fed CTRL (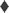), TRP2 (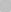) or TRP4 (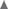), and intraperitoneally injected with *Phdp* (*n* = 10).

Mortalities in LD fish started 5 days after infection in the CTRL group, while fish fed TRP4 started to die at day 6 post-injection; no mortalities were registered in fish fed TRP2 (Figure 1A). At the end of the challenge trial, cumulative mortality in fish fed CTRL was 30% reaching its maximum value at day 6, while fish fed TRP4 reached 80% of mortalities which were extended from the 14th day after injection until the end of the experiment.

At HD, mortality in fish fed CTRL presented a similar response to that observed in fish fed the same diet at LD (30% cumulative mortality, Figure 1B). In contrast, mortality increased to 10% in fish fed TRP2 and decreased also to 10% in fish fed TRP4, although a delay over time (5 days) was observed in TRP2 with respect to the TRP4 group.

### Hematology

Regardless of rearing density, fish fed TRP4 showed higher Hb content than fish fed TRP2, but no differences were observed compared to fish fed CTRL (Table 5). At Fish maintained at LD did not show differences in MCV between treatments, while at HD fish fed TRP4 presented higher MCV than fish fed CTRL and TRP2 (Table 5). MCV of fish fed TRP4 was also higher at HD than that of their counterparts under LD condition. At LD there were also no differences in MCH between groups, while at HD it was also higher in fish fed TRP4 than TRP2. In fish held at HD, MCH was higher in fish fed TRP4 and the CTRL than their LD counterparts. No differences were observed regarding Ht and MCHC (Table 5).

**Table 5:** Hematocrit (Ht), Hb, mean corpuscular volume (MCV), mean corpuscular hemoglobin (MCH), and mean corpuscular hemoglobin concentration (MCHC) in Senegalese sole after 38 days held at different treatments.

Parameters	Treatments					
	LD			HD		
	CTRL	TRP2	TRP4	CTRL	TRP2	TRP4
Ht (%)	13.6 ± 4.3	11.7 ± 1.3	12.5 ± 2.0	11.8 ± 1.9	12.7 ± 2.2	13.2 ± 1.9
Hb (g dL^−1^)	2.7 ± 0.5	2.5 ± 0.5	2.9 ± 0.6	2.5 ± 0.8	2.4 ± 0.6	2.9 ± 0.6
MCV (μm^3^)	94.4 ± 34.9	105.3 ± 25.5	110.4 ± 23.8^∗^	130.0 ± 32.6 a	122.7 ± 13.6 a	180.7 ± 16.7 b^#^
MCH (pg cell^−1^)	22.2 ± 8.7^∗^	25.8 ± 4.8	25.0 ± 3.4^∗^	34.2 ± 4.1 ab^#^	22.3 ± 8.3 a	39.4 ± 8.6 b^#^
MCHC (g 100 mL^−1^)	22.4 ± 3.0	22.5 ± 2.6	24.1 ± 3.9	21.8 ± 8.8	18.6 ± 5.7	22.7 ± 4.1

Parameters	Density	Diet	Density × diet	CTRL	TRP2	TRP4
Ht	ns	ns	ns			
Hb	ns	0.022	ns	ab	a	b
MCV	<0.001	0.002	0.035			
MCH	<0.001	0.012	0.003			
MCHC	ns	ns	ns			

*Values are expressed as means ± SD (n = 12). Different letters mean significant differences between dietary treatments. Different symbols denote significant differences between densities (p < 0.05), whereas ns means non-significant differences. Two-way ANOVA; Tukey post hoc test; p ≤ 0.05.*

**FIGURE 2:**
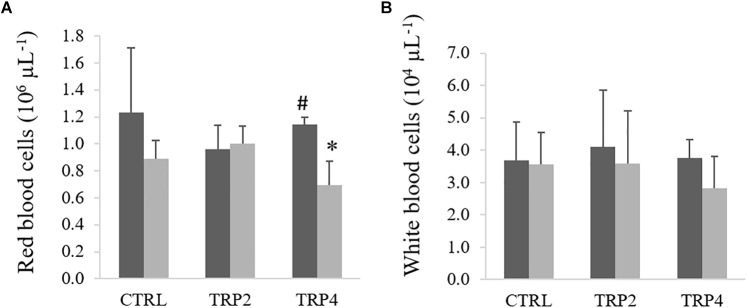
Red blood cells **(A)** and total white blood cells **(B)** in Senegalese sole held at low (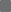) or high (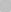) stocking densities and fed dietary treatments for 38 days (mean ± SD, *n* = 9). Different symbols indicate significant differences attributed to stocking density. Two-way ANOVA; Tukey *post hoc* test; *p* ≤ 0.05.

**FIGURE 3:**
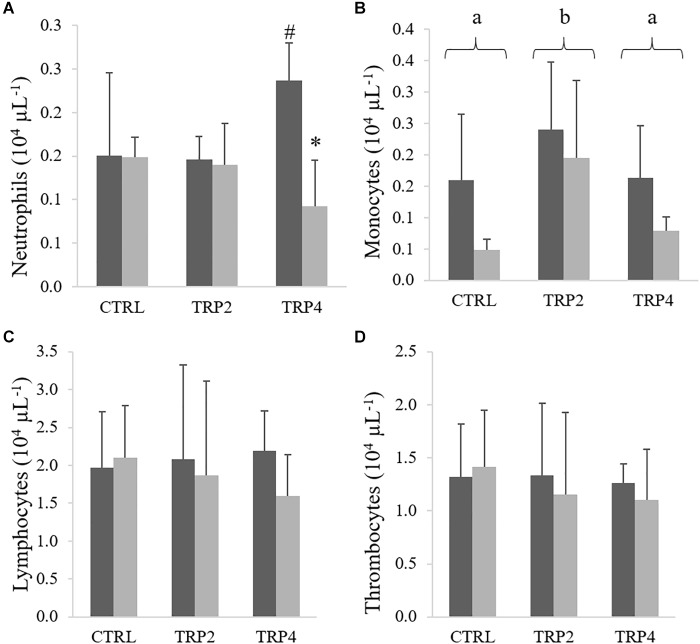
Neutrophils **(A)**, monocytes **(B)**, lymphocytes **(C)**, and thrombocytes **(D)** in Senegalese sole held at low (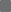) or high (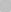) stocking densities and fed dietary treatments for 38 days (mean ± SD, *n* = 9). Different symbols indicate significant differences attributed to stocking density. Different letters stand for significant differences between dietary treatments. Two-way ANOVA; Tukey *post hoc* test; *p* ≤ 0.05.

In fish fed TRP4, peripheral RBC were lower in fish held at HD than at LD, no other differences being observed between diets or rearing densities (Figure 2A). No effects of diet or rearing density were detected on total WBC (Figure 2B). In contrast, peripheral neutrophils concentration decreased in TRP4-fed fish under HD compared to their counterparts held at LD, but no other differences were observed among groups (Figure 3A). Regarding circulating monocytes, numbers were higher in fish fed TRP2 than in fish fed CTRL and TRP4, irrespective of rearing density (Figure 3B). Finally, lymphocytes and thrombocyte concentrations were not significantly affected by experimental treatments (Figure 3C,D, respectively).

### Plasma Cortisol and Metabolites

**FIGURE 4:**
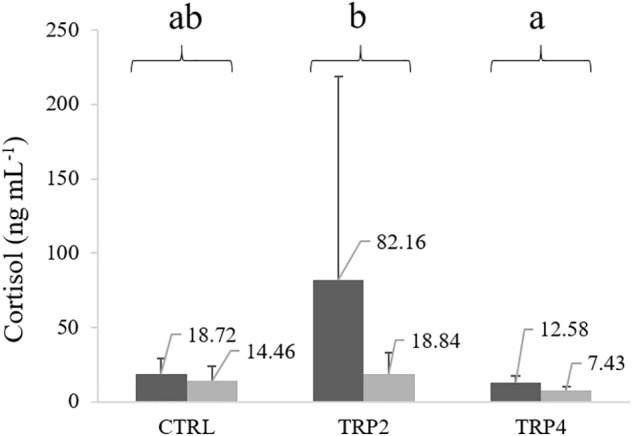
Plasma cortisol levels in Senegalese sole held at low (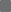) or high (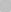) stocking densities and fed dietary treatments for 38 days (mean ± SD, *n* = 12). Values above each column are cortisol mean concentrations. Different letters stand for significant differences between dietary treatments. Two-way ANOVA; Tukey *post hoc* test; *p* ≤ 0.05.

Plasma cortisol levels were lower in fish held at HD than at LD, regardless of dietary treatment. Regarding dietary treatments, fish fed TRP4 showed lower plasma cortisol concentration compared to fish fed TRP2, but no significant differences were observed compared to CTRL-fed fish due to high variability in fish fed TRP2 (Figure 4).

**FIGURE 5:**
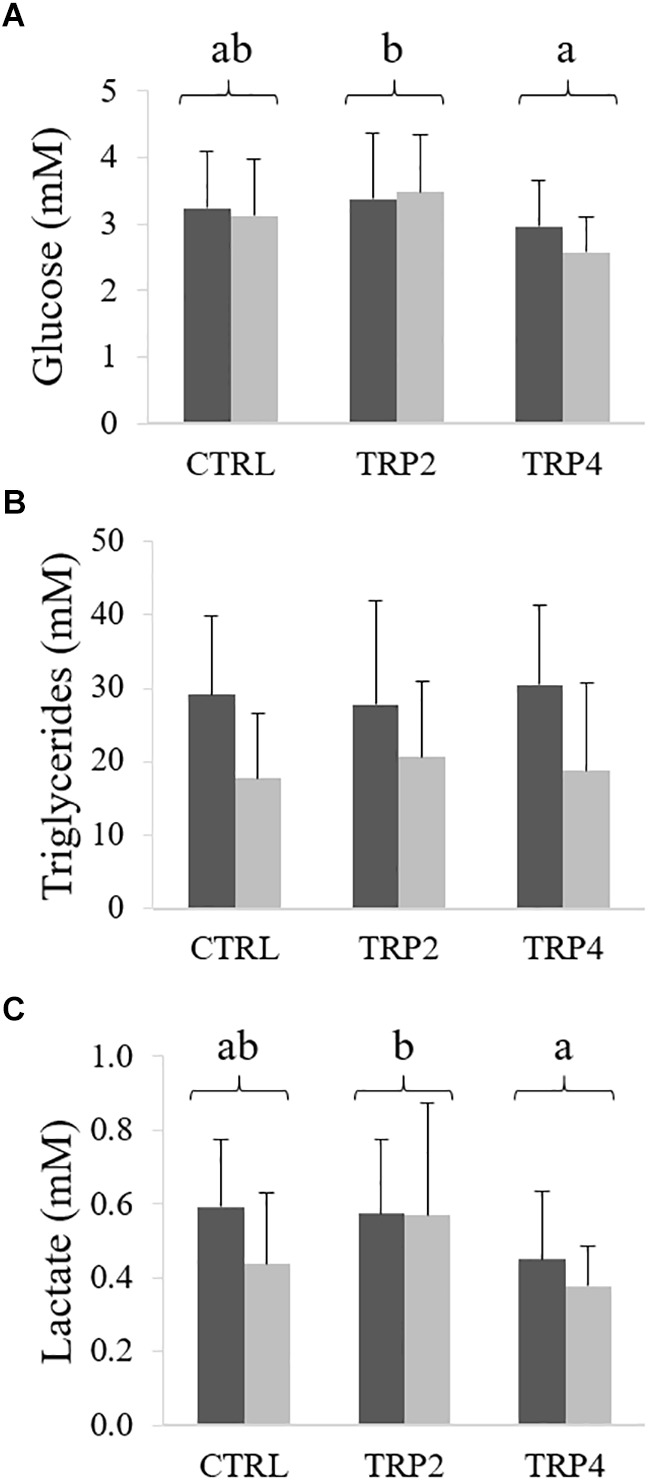
Plasma glucose **(A)**, lactate **(B)**, and triglycerides **(C)** levels in Senegalese sole held at low (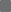) or high (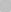) stocking densities and fed dietary treatments for 38 days (mean ± SD, *n* = 12). Different letters stand for significant differences between dietary treatments. Two-way ANOVA; Tukey *post hoc* test; *p* ≤ 0.05.

Plasma glucose and lactate levels were lower in fish fed TRP4 than TRP2, regardless rearing density (Figure 5A,C, respectively). Differently, dietary treatment did not affect plasma triglycerides, while levels were lower in fish held at HD (Figure 5B).

### Innate Humoral Parameters

**FIGURE 6:**
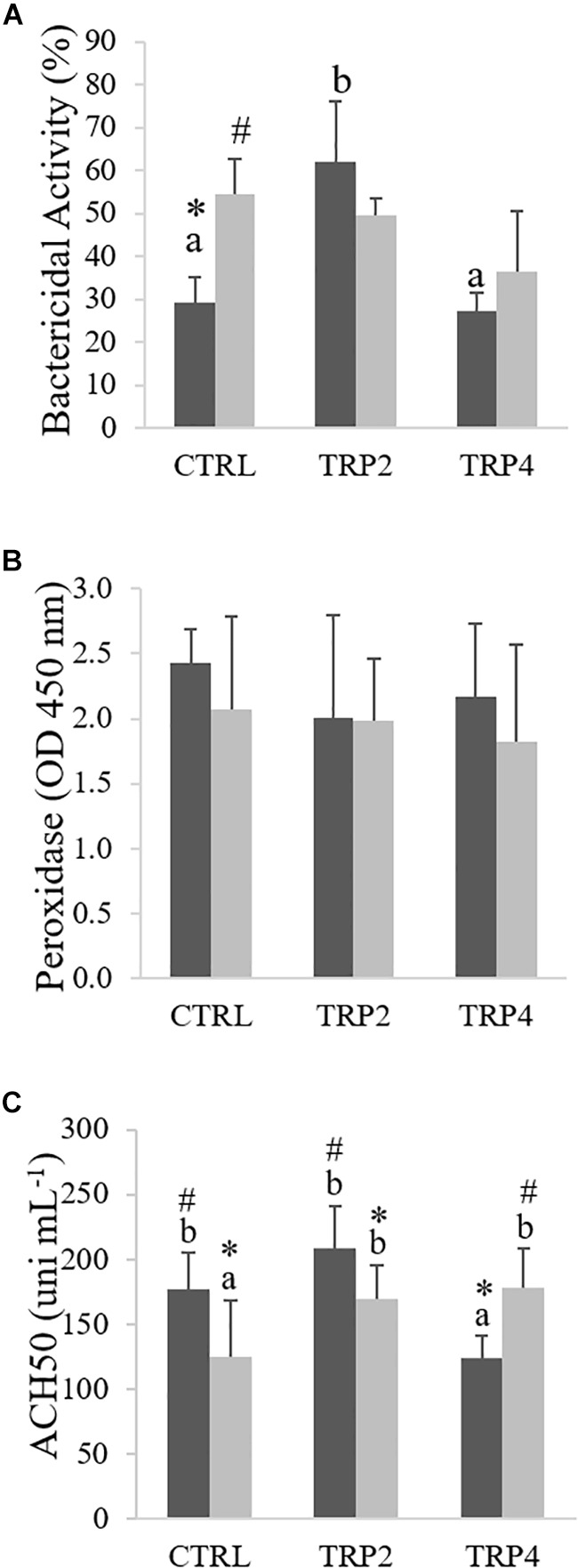
Plasma total bactericidal activity **(A)**, peroxidase **(B)**, and ACH50 activity **(C)** in Senegalese sole held at low (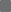) or high (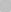) stocking densities and fed dietary treatments for 38 days (mean ± SD, *n* = 12). Different symbols indicate significant differences attributed to stocking density. Different letters stand for significant differences between dietary treatments. Two-way ANOVA; Tukey *post hoc* test; *p* ≤ 0.05.

Plasma bactericidal activity increased in fish fed TRP2 and held at LD compared to fish fed CTRL or TRP4 under the same density conditions, whereas dietary treatments did not affect the bactericidal activity of Senegalese sole held at HD (Figure 6A). Still, a significant interaction was observed in fish fed the CTRL diet which translated in an enhanced bactericidal activity in fish held at HD compared to their counterparts reared at LD (Figure 6A). On the other hand, neither dietary treatments, nor rearing density significantly affected plasma peroxidase levels (Figure 6B). Regarding plasma ACH50 levels, interactive effects between density and dietary treatments were observed, in which fish fed CTRL and TRP2 held at LD presented higher ACH50 activity than those maintained at HD (Figure 6C). Differently, ACH50 was higher in fish fed TRP4 held at HD compared to those at LD. Moreover, TRP2-fed fish showed higher ACH50 activity than those fed TRP4 under LD condition, while at HD, both supplemented diets enhanced ACH50 activity compared to those fed CTRL.

### Gene Expression

**FIGURE 7:**
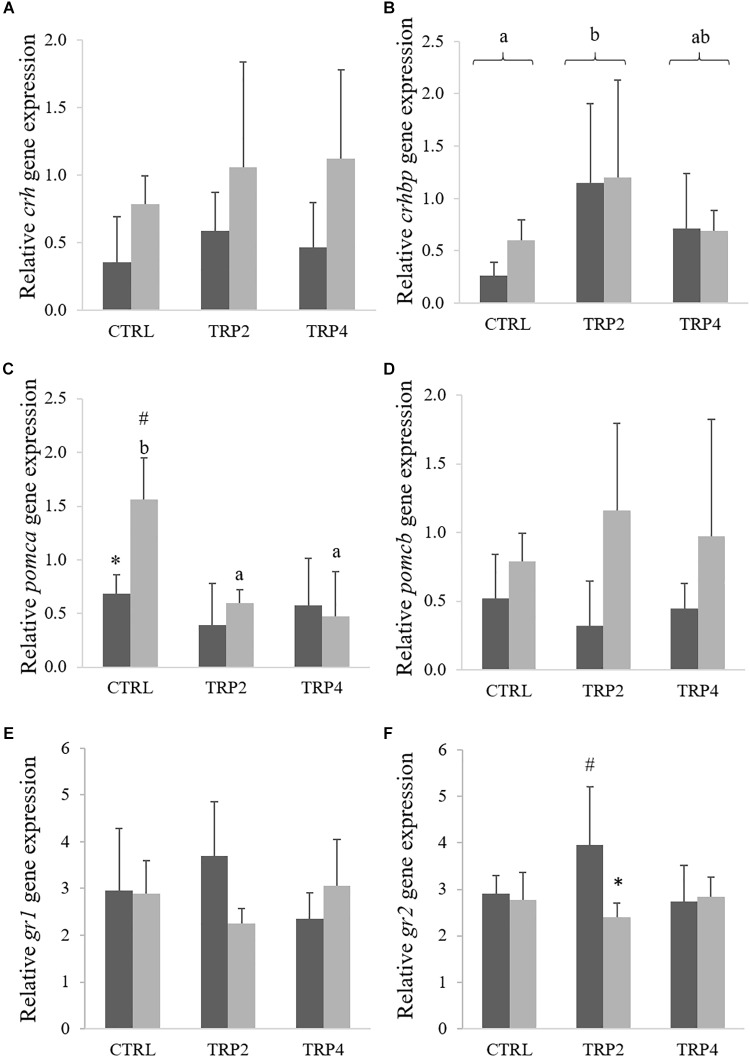
Corticotropin-releasing hormone (*crh*, **A**), corticotropin-releasing hormone-binding protein (*crhbp*, **B**), proopiomelanocortin A (*pomca*, **C**) and B (*pomcb*, **D**), and glucocorticoid receptor 1 (*gr1*, **E**) and 2 (*gr2*, **F**) in Senegalese sole held at low (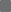) or high (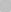) stocking densities and fed dietary treatments for 38 days (mean ± SD, *n* = 6). Different symbols indicate significant differences attributed to stocking density. Different letters stand for significant differences between dietary treatments. Two-way ANOVA; Tukey *post hoc* test; *p* ≤ 0.05.

Expression for *crh* was not affected by diet composition, but increased in fish held at HD regardless dietary treatments (Figure 7A), whereas *crhbp* expression level was higher in TRP2-fed fish than in CTRL-fed fish regardless stocking density conditions (Figure 7B). Expression for *pomca* was down-regulated in fish fed TRP2 and TRP4 under HD condition compared to CTRL-fed animals, whereas fish held at HD and fed CTRL also presented higher *pomca* transcripts respect to their counterparts at LD (Figure 7C). Moreover, HD conditions increased *pomcb* gene expression relatively to LD, while no diet-mediated effects were observed (Figure 7D). Dietary treatments did not affect *gr1* transcript levels (Figure 7E), which contrasted a down-regulation of *gr2* gene expression in TRP2-fed fish at HD compared to their LD counterparts (Figure 7F).

## Discussion

High stocking density conditions are used in intensive fish farms and though hardly unavoidable, they are regarded as one of intensive aquaculture production biggest bottlenecks, as it represents an important stress-inducing factor (Saeij et al., 2003; Varsamos et al., 2006; Aedo et al., 2015). Amongst other negative effects, growth, immunity, and disease resistance are known to be severely compromised in fish undergoing chronic stress (Tort, 2011). In the present study, fish held at HD decreased growth performance (i.e., FBW and SGR) even though VFI was enhanced, in line to that described for other flatfish species such as common sole *Solea solea* (Schram et al., 2006), turbot *Scophthalmus maximus* (Irwin et al., 1999), or California halibut *Paralichthys californicus* (Merino et al., 2007). This tertiary stress response has already been observed in many other chronically stressed fish (Bonga, 1997), including the Senegalese sole (Arjona et al., 2009). However, results from this study contrast previous studies, which suggested that Senegalese sole cope well with high stocking densities with no implications for growth under both experimental and farming conditions (Salas-Leiton et al., 2008; Costas et al., 2013b; Andrade et al., 2015). This apparent discrepancy could be related to differences among developmental stages, rearing conditions or genetic factors (Barton, 2002), which may have interfered in the typical coping style described for Senegalese sole (Costas et al., 2008; Andrade et al., 2015). In fact, rearing tanks (i.e., shape and size) from the present study are likely to have negatively influenced fish growth performance. This is an important issue to have into account in future studies with a clear impact/knowledge transfer to the industry.

Cortisol, considered the most direct evidence of stress in fish (Flik et al., 2006), was relatively high in fish fed CTRL and kept under LD condition compared to levels measured in Senegalese sole in control conditions in previous studies (Costas et al., 2008, 2013b; Salas-Leiton et al., 2010; Wunderink et al., 2011). Still, we should bear in mind that both cortisol levels and density conditions from fish fed CTRL at LD from the present study are comparable to those held at high density by most of the mentioned studies. Moreover, the fact that CTRL-fed fish held at HD did not show higher plasma cortisol than their LD counterparts might be related to negative feedback mechanisms established in the HPI axis, as a strategy of chronically stressed animals to attenuate an exacerbated stress response (Bonga, 1997; Mommsen et al., 1999).

Though not significantly different from CTRL-fed fish, plasma cortisol concentrations were comparatively higher in TRP2-fed fish compared to TRP4. These cortisol patterns might be related to the tryptophan role in the central nervous system as an exclusive precursor of serotonin. Neuronal serotonin concentration is expected to be higher upon increased availability of its precursor (tryptophan), as tryptophan hydroxylase is not saturated at tryptophan physiological levels (Herrero et al., 2007; Azeredo et al., 2017; Hoseini et al., 2017). Furthermore, serotonin is partly responsible for either inhibiting or stimulating pathways in central nervous system (Spinedi and Negrovilar, 1983) as previously observed in rainbow trout (Winberg and Lepage, 1998; Winberg et al., 2001; Lepage et al., 2002). This might therefore be the mechanism explaining the increasing trend in cortisol secretion in TRP2-fed fish, even though fish were in LD stocking conditions. In providing a tryptophan surplus, serotonin production was enhanced inducing an endocrine response in these fish. These results are in accordance with those obtained in rainbow trout, which cortisol levels increased with graded inclusion of dietary tryptophan in non-stressed fish while decreased in stressed animals (Lepage et al., 2002, 2003). However, in the present study, such gradual increase of tryptophan did not similarly induce cortisol production, as TRP4-fed fish did not present higher cortisol concentration than those fed TRP2. This impairment of cortisol response, together with the observed increased disease susceptibility might be pointing a possible toxic effect of the highest supplementation level of tryptophan, which in turn could produce a clear misbalance for the correct functioning of the HPI axis, masking or desensitizing the stress response at peripheral level. At HD stocking conditions, it is expected that a neuroendocrine response is developed in which increased cortisol production is usually observed. Present results corroborate once more the aforementioned studies, as Senegalese sole reared at HD and fed tryptophan supplemented diets produced cortisol in lower (TRP2-fed fish) or similar (TRP4-fed fish) amounts than their LD counterparts. This apparent adaptation to a stressful situation might again have been mediated by serotonin neuronal intervention, which is known to inhibit pituitary ACTH secretion during an ongoing neuroendocrine response (Höglund et al., 2002).

Since tryptophan – through its metabolite serotonin – has a direct impact on the central nervous system (Höglund et al., 2005, 2007; Gesto et al., 2013), brain mediators of stress response are expected to be modulated as well. The involvement of serotonin in the neuroendocrine regulation of the stress response seems to be similar within the vertebrate linage sine serotonin plays a role in the control of the HPI axis in fish, mainly through its effects on the release of corticotropin-releasing factor from the hypothalamus (Winberg et al., 1997). In the present study, stressful conditions potentiated an increase of *crh* expression that was transversal to all dietary treatments, while tryptophan dietary supplementation did not seem to affect Crh production, at least at a molecular level. Yet, *crhbp* gene expression did not follow the same response and was unaffected by stocking conditions in fish held in HD. Moreover, up-regulation of *crhbp* in fish fed TRP2 relatively to CTRL fish suggests that nutritional modulation of the HPI axis might start as high as at the hypothalamus. The mRNA from *crhbp* is translated to a protein known to regulate Crh availability and activity. Regulatory mechanisms might both prolong Crh half-life, as by binding to Crh prevents it from being degraded, and reduce Crh activity by sequestering ligand from receptor (Seasholtz et al., 2002; Moltesen et al., 2016). In spite of *crh* not differing between dietary treatments, the increased expression of *crhbp* in TRP2-fed fish points at tryptophan higher availability as a key modulator of the HPI axis activity.

An even clearer aspect of tryptophan neuroendocrine modulation is the *pomca* down-regulation in fish held at HD and fed TRP2 or TRP4, relative to CTRL. Both *pomca* and *pomcb* are structurally mostly identical, encoding several bioactive peptide hormones such as ACTH, β-MSH, and β-endorphin (Navarro et al., 2016). However, the two paralogs have distinct cleavage sites, thereby each yielding different hormones; *pomca* seems to translate to functional ACTH, while *pomcb* gives rise to β-MSH (Wunderink et al., 2012). Accordingly, present results show that *pomca*, and not *pomcb*, is modulated by stress, as fish fed CTRL showed a rise in *pomca* mRNA levels when held at HD stocking conditions. In contrast, dietary tryptophan prevented such increase in *pomca* transcripts in fish held at HD, possibly denoting tryptophan indirect modulation of the HPI axis activity. Indeed, *pomca* mRNA levels in HD fish fed TRP2 and TRP4 seem correlated (*R*^2^ = 0.67, *P* = 0.018) with a decreasing trend in plasma cortisol levels in fish fed dietary tryptophan supplements. Despite not as elucidating, results on the expression of cortisol receptor *gr2* seem to corroborate those of cortisol itself and of *pomca* as its expression is down-regulated in TRP2-fed fish held at HD compared to their LD counterparts.

In the present study, available data may suggest a possible relationship between increased serotonin and *pomca* mRNA levels in fish reared in HD in which the tryptophan supplementation was effective in reducing the stress response. For instance, it is known that pharmacological blockade of serotonin receptors increases the expression of POMC in rainbow trout (Pérez-Maceira et al., 2016). It is tempting to speculate that the study pointed to an implication of brain serotonin in some of the effects previously described in stressed fish and those fish supplemented with tryptophan, and reinforces the need to further explore the links between tryptophan nutrition, serotonin synthesis and the implication of the HPI axis in stressed fish.

Glucose and triglycerides stores mobilization and glucose anaerobic metabolism are activated whenever a stress response is triggered and, therefore, are considered part of the secondary stress response (Bonga, 1997). Accordingly, glucose plasma levels were observed to increase along with cortisol as a response to chronic stress (Costas et al., 2008, 2013b). Differently, in the present study, glucose concentration was not affected by high stocking density and a similar pattern was observed in both great and Siberian sturgeon, *Huso huso* and *Acipenser baerii*, respectively, exposed to similar chronic stress conditions (Rafatnezhad et al., 2008; Hasanalipour et al., 2013). Triglycerides levels were lower in plasma of fish held at HD than in those in LD conditions, as previously observed by Yarahmadi et al. (2015) in rainbow trout held at high density for 30 days. Lower triglycerides could possibly be a direct consequence of lower feed intake known to be related to stressful holding conditions as previously observed in the Atlantic cod, *Gadus morhua* (Lambert and Dutil, 2001). This, together with the observed lower HSI in HD groups and the fact that no such differences were observed either in glucose nor in lactate levels, might be a signal of total energy substrate exhaustion in fish undergoing a challenging, chronic stress response.

Cortisol is known to modulate several physiological responses, including immune responses. Thus, if an acute stress might be a trigger of immunological mechanisms either directly or via the HPI axis (Tort, 2011), chronic stress conditions have long been associated with immunosuppression and general poor welfare in fish (Tort, 2011; Gadan et al., 2012; Philip et al., 2012). Neither dietary treatments nor stocking density seemed to affect total WBC. However, fish reared at HD and fed TRP4 had decreased number of RBC and increased erythrocyte volume (MCV index) and hemoglobin content (MCH) compared to their LD counterparts, which might be a compensatory strategy to ensure oxygen transport (Robb and Abrahams, 2003). As no direct connection is known to exist between tryptophan and erythrocyte oxygen transport, further research is required to unveil the biological mechanisms associated to stress response that are triggering these hematological changes.

Indoleamine 2,3-dioxygenase-mediated tryptophan catabolism is known to modulate T-cell proliferation and impair other immune cells function (Frumento et al., 2001; Moffett and Namboodiri, 2003). Yet, its activity is only triggered with immune stimulation in both teleosts and mammals (Munn and Mellor, 2013; Cortes et al., 2016). Although present results are only related to general immune status (i.e., in the absence of immune stimulation), stressful conditions and the inherent HPI axis activation are likely to activate other immune mechanisms, in light of what is known about regulatory pathways between immune and neuroendocrine systems (Verburg-Van Kemenade et al., 2011). This bi-directional communication between immune and neuroendocrine systems was perhaps what led total bactericidal activity to increase in fish fed CTRL held at HD. Hence, indoleamine 2,3-dioxygenase cannot be excluded as possible mediator of changes in cell counts. Though not statistically significant, neutrophil concentration in TRP4-fed fish held at LD tended to be higher than in CTRL-fed fish, which could give cues about the immune system activation produced during the days previous to the end of the trial (with the subsequent stabilization of mortality rates), or even about the mediated orchestration to further induce a refractory answer during infection in a longer response. Interestingly, this apparent neutrophilia, together with higher monocyte concentration in TRP2-fed fish, is in opposite direction from what is expected of indoleamine 2,3-dioxygenase action.

Peroxidase activity is a signal of cell activation and it was not significantly affected in this study, suggesting that increased tryptophan availability did not enhance cell activation, even though improved cell recruitment. Moreover, effects of tryptophan supplementation on cell response are quite different from those on plasma ACH50 levels and on disease resistance. ACH50 plays an important role in the innate immune machinery, being responsible for pathogen lysis and opsonization (Nonaka and Smith, 2000), and in the present study it varied in line with the ability of fish to overcome infection. ACH50 was inhibited in fish fed TRP4 and held at LD. However, while ACH50 levels decreased in fish fed CTRL and under HD condition, dietary tryptophan supplementation not only counteracted this stress-induced decrease, but enhanced plasma ACH50 activity in fish held at HD. Similar results were previously reported by Hoseini et al. (2016), in Persian sturgeon, *Acipenser persicus*, fed tryptophan-supplemented diets and subjected to an acute stress.

Dietary tryptophan supplementation seemed to translate into a double-edged sword in Senegalese sole reared at LD since it induced opposite effects in terms of immune responses and disease resistance. On the one hand, fish fed TRP2 showed increased plasma bactericidal and ACH50 activities as well as circulating monocyte numbers, which translated in no mortalities after a bacterial challenge. On the other hand, despite it did not significantly affect other immune parameters, TRP4 decreased plasma ACH50 activity and led to the highest cumulative mortalities among experimental treatments. These data are indeed intriguing and suggest further experiments to unravel tryptophan role during a disease challenge. Although the contrasting responses appear not to be mediated by the HPI axis, since fish reared at LD and fed both tryptophan treatments showed similar responses, more genes linked to synthesis and release would be of assistance. Whether the observed increased disease susceptibility in fish fed TRP4 and held at LD is mediated by indoleamine 2,3-dioxygenase-related mechanisms needs to be further explored.

In contrast, it has been reported that tryptophan needs to increase in Senegalese sole reared under high density conditions (Costas et al., 2008). In the present study, both dietary tryptophan treatments seemed to compensate this increase demand, which translated in lower *pomca* transcripts, plasma cortisol levels, enhanced immune status and disease resistance. In this context, dietary tryptophan surplus as treatment to counteract the negative effects of chronic stress seems to be realistic for Senegalese sole.

In summary, this study suggested that tryptophan dietary treatment could be a promising strategy to counteract chronic stress-induced immunosuppression in Senegalese sole reared at high stocking density. In fish reared at low density, dietary tryptophan supplementation above requirements seems to be both beneficial and harmful, depending on the level of supplementation, an effect that remains to be unraveled. Furthermore, both nature and duration of stress inflicted in fish might be determinant while evaluating tryptophan potential in modulating the neuroendocrine response.

## Ethics Statement

This study was carried out by trained scientists in full compliance with national rules and following the European Directive 2010/63/EU of the European Parliament and the European Union Council on the protection of animals used for scientific purposes. The protocol was approved by the Animal Welfare Committee of the Interdisciplinary Centre of Marine and Environmental Research and carried out in a registered installation (N16091.UDER).

## Author Contributions

RA, MM, and JM conducted the main experimental work and performed all humoral and cellular analysis. RA wrote the manuscript under the supervision of BC. JM-S and GM-R analyzed the plasma cortisol and metabolites, and performed the brain and hypophyseal gene expression analyses. HP was responsible for dietary formulation and determined amino acid composition of the experimental diets. AO-T, AA, JMM, and BC conceived the experiments and contributed with both reagents and goods. All authors contributed to and approved the manuscript.

## Conflict of Interest Statement

The authors declare that the research was conducted in the absence of any commercial or financial relationships that could be construed as a potential conflict of interest.

## References

[B1] Aedo J. E. Maldonado J. Aballai V. Estrada J. M. Bastias-Molina M. Meneses C. (2015). mRNA-seq reveals skeletal muscle atrophy in response to handling stress in a marine teleost, the red cusk-eel (*Genypterus chilensis*). *BMC Genomics* 16:1024. 10.1186/s12864-015-2232-7 26626593 PMC4667402

[B2] Afonso A. Lousada S. Silva J. Ellis A. E. Silva M. T. (1998). Neutrophil and macrophage responses to inflammation in the peritoneal cavity of rainbow trout *Oncorhynchus mykiss*. A light and electron microscopic cytochemical study. *Dis. Aquat. Organ.* 34 27–37. 10.3354/Dao034027 9867437

[B3] Andrade T. Afonso A. Perez-Jimenez A. Oliva-Teles A. de las Heras V. Mancera J. M. (2015). Evaluation of different stocking densities in a Senegalese sole (*Solea senegalensis*) farm: implications for growth, humoral immune parameters and oxidative status. *Aquaculture* 438 6–11. 10.1016/j.aquaculture.2014.12.034

[B4] AOAC (2000). *Official Methods of Analysis.* Washington, DC: Association of Official Analytical Chemists, 1018.

[B5] Arends R. J. Mancera J. M. Munoz J. L. Bonga S. E. W. Flik G. (1999). The stress response of the gilthead sea bream (*Sparus aurata* L.) to air exposure and confinement. *J. Endocrinol.* 163 149–157. 10.1677/joe.0.1630149 10495417

[B6] Arijo S. Chabrillon M. Diaz-Rosales P. Rico R. M. Martinez-Manzanares E. Balebona M. C. (2005). Bacteria isolated from outbreaks affecting cultured sole, *Solea senegalensis* (Kaup). *Bull. Eur. Assoc. Fish Pathol.* 25 148–154.

[B7] Arjona F. J. Vargas-Chacoff L. Ruiz-Jarabo I. Goncalves O. Pascoa I. del Rio M. P. M. (2009). Tertiary stress responses in Senegalese sole (*Solea senegalensis* Kaup, 1858) to osmotic challenge: implications for osmoregulation, energy metabolism and growth. *Aquaculture.* 287 419–426. 10.1016/j.aquaculture.2008.10.047

[B8] Azeredo R. Machado M. Afonso A. Fierro-Castro C. Reyes-López F. E. Tort L. (2017). Neuroendocrine and immune responses undertake different fates following tryptophan or methionine dietary treatment: tales from a teleost model. *Front. Immunol.* 8:1226. 10.3389/fimmu.2017.01226 29021795 PMC5623689

[B9] Barton B. A. (2002). Stress in fishes: a diversity of responses with particular reference to changes in circulating corticosteroids. *Integr. Comp. Biol.* 42 517–525. 10.1093/icb/42.3.517 21708747

[B10] Bonga S. E. W. (1997). The stress response in fish. *Physiol. Rev.* 77 591–625. 9234959 10.1152/physrev.1997.77.3.591

[B11] Cortes J. Alvarez C. Santana P. Torres E. Mercado L. (2016). Indoleamine 2,3-dioxygenase: first evidence of expression in rainbow trout (*Oncorhynchus mykiss*). *Dev. Comp. Immunol.* 65 73–78. 10.1016/j.dci.2016.06.020 27370975

[B12] Costas B. Aragao C. Dias J. Afonso A. Conceicao L. E. C. (2013a). Interactive effects of a high-quality protein diet and high stocking density on the stress response and some innate immune parameters of Senegalese sole *Solea senegalensis*. *Fish Physiol. Biochem.* 39 1141–1151. 10.1007/s10695-013-9770-1 23341074

[B13] Costas B. Rego P. C. Simoes I. Marques J. F. Castro-Cunha M. Afonso A. (2013b). Cellular and humoral immune responses of Senegalese sole, *Solea senegalensis* (Kaup), following challenge with two *Photobacterium damselae* subsp piscicida strains from different geographical origins. *J. Fish Dis.* 36 543–553. 10.1111/Jfd.12033 23163607

[B14] Costas B. Aragao C. Mancera J. M. Dinis M. T. Conceicao L. E. C. (2008). High stocking density induces crowding stress and affects amino acid metabolism in Senegalese sole *Solea senegalensis* (Kaup 1858) juveniles. *Aquac. Res.* 39 1–9. 10.1111/j.1365-2109.2007.01845.x

[B15] Costas B. Aragao C. Soengas J. L. Miguez J. M. Rema P. Dias J. (2012). Effects of dietary amino acids and repeated handling on stress response and brain monoaminergic neurotransmitters in Senegalese sole (*Solea senegalensis*) juveniles. *Comp. Biochem. Phys. A.* 161 18–26. 10.1016/j.cbpa.2011.08.014 21903174

[B16] Costas B. Conceicao L. E. C. Dias J. Novoa B. Figueras A. Afonso A. (2011). Dietary arginine and repeated handling increase disease resistance and modulate innate immune mechanisms of Senegalese sole (*Solea senegalensis* Kaup, 1858). *Fish Shellfish Immun.* 31 838–847. 10.1016/j.fsi.2011.07.024 21820517

[B17] De Vries J. W. Koski C. M. Egberg D. C. Larson P. A. (1980). Comparison between a spectrophotometric and a high-pressure liquid chromatography method for determining tryptophan in food products. *J. Agric. Food Chem.* 28 896–898. 10.1021/jf60231a025 7462515

[B18] Flik G. Klaren P. H. M. Van den Burg E. H. Metz J. R. Huising M. O. (2006). CRF and stress in fish. *Gen. Comp. Endocrinol.* 146 36–44. 10.1016/j.ygcen.2005.11.005 16403502

[B19] Frumento G. Rotondo R. Tonetti M. Ferrara G. B. (2001). T cell proliferation is blocked by indoleamine 2,3-dioxygenase. *Transplant. Proc.* 33 428–430. 10.1016/s0041-1345(00)02078-9 11266894

[B20] Gadan K. Marjara I. S. Sundh H. Sundell K. Evensen O. (2012). Slow release cortisol implants result in impaired innate immune responses and higher infection prevalence following experimental challenge with infectious pancreatic necrosis virus in Atlantic salmon (*Salmo salar*) parr. *Fish Shellfish Immun.* 32 637–644. 10.1016/j.fsi.2012.01.004 22281610

[B21] Gesto M. Lopez-Patino M. A. Hernandez J. Soengas J. L. Miguez J. M. (2013). The response of brain serotonergic and dopaminergic systems to an acute stressor in rainbow trout: a time course study. *J. Exp. Biol.* 216 4435–4442. 10.1242/Jeb.091751 24031060

[B22] Graham S. Jeffries A. H. Secombes C. J. (1988). A novel assay to detect macrophage bactericidal activity in fish: factors influencing the killing of *Aeromonas salmonicida*. *J. Fish Dis.* 11 389–396. 10.1111/j.1365-2761.1988.tb00734.x

[B23] Hasanalipour A. Eagderi S. Poorbagher H. Bahmani M. (2013). Effects of stocking density on blood cortisol, glucose and cholesterol levels of immature siberian sturgeon (*Acipenser baerii* Brandt, 1869). *Turk. J. Fish. Aquat. Sci.* 13 27–32. 10.4194/1303-2712-v13_1_04

[B24] Haukenes A. H. Barton B. A. (2004). Characterization of the cortisol response following an acute challenge with lipopolysaccharide in yellow perch and the influence of rearing density. *J. Fish Biol.* 64 851–862. 10.1111/j.1095-8649.2004.00354.x

[B25] Herrero M. J. Martinez F. J. Miguez J. M. Madrid J. A. (2007). Response of plasma and gastrointestinal melatonin, plasma cortisol and activity rhythms of European sea bass (*Dicentrarchus labrax*) to dietary supplementation with tryptophan and melatonin. *J. Comp. Physiol. B.* 177 319–326. 10.1007/s00360-006-0131-6 17123089

[B26] Höglund E. Bakke M. J. Øverli Ø Winberg S. Nilsson G. E. (2005). Suppression of aggressive behaviour in juvenile Atlantic cod (*Gadus morhua*) by L-tryptophan supplementation. *Aquaculture* 249 525–531. 10.1016/j.aquaculture.2005.04.028

[B27] Höglund E. Balm P. H. M. Winberg S. (2002). Stimulatory and inhibitory effects of 5-HT1A receptors on adrenocorticotropic hormone and cortisol secretion in a teleost fish, the Arctic charr (*Salvelinus alpinus*). *Neurosci. Lett.* 324 193–196. 10.1016/S0304-3940(02)00200-8 12009521

[B28] Höglund E. Øverli,Ø and Winberg S. (2019). Tryptophan metabolic pathways and brain serotonergic activity: a comparative review. *Front. Endocrinol.* 10:158 10.3389/fendo.2019.00158 PMC6463810 31024440

[B29] Höglund E. Sorensen C. Bakke M. J. Nilsson G. E. Øverli Ø (2007). Attenuation of stress-induced anorexia in brown trout (*Salmo trutta*) by pre-treatment with dietary L-tryptophan. *Br. J. Nutr.* 97 786–789. 10.1017/S0007114507450280 17349093

[B30] Hoseini S. M. Mirghaed A. T. Mazandarani M. Zoheiri F. (2016). Serum cortisol, glucose, thyroid hormones’ and non-specific immune responses of Persian sturgeon, *Acipenser persicus* to exogenous tryptophan and acute stress. *Aquaculture* 462 17–23. 10.1016/j.aquaculture.2016.04.031

[B31] Hoseini S. M. Pérez-Jiménez A. Costas B. Azeredo R. Gesto M. (2017). Physiological roles of tryptophan in teleosts: current knowledge and perspectives for future studies. *Rev. Aquacult.* 11 3–24. 10.1111/raq.12223

[B32] Irwin S. O’Halloran J. FitzGerald R. D. (1999). Stocking density, growth and growth variation in juvenile turbot, *Scophthalmus maximus* (Rafinesque). *Aquaculture* 178 77–88. 10.1016/S0044-8486(99)00122-2

[B33] Kaushik S. J. (1998). Whole body amino acid composition of European seabass (*Dicentrarchus labrax*), gilthead seabream (*Sparus aurata*) and turbot (*Psetta maxima*) with an estimation of their IAA requirement profiles. *Aquat. Living Resour.* 11 355–358. 10.1016/S0990-7440(98)80007-7

[B34] Lambert Y. Dutil J. D. (2001). Food intake and growth of adult Atlantic cod (*Gadus morhua* L.) reared under different conditions of stocking density, feeding frequency and size-grading. *Aquaculture* 192 233–247. 10.1016/S0044-8486(00)00448-8

[B35] Lepage O. Tottmar O. Winberg S. (2002). Elevated dietary intake of L-tryptophan counteracts the stress-induced elevation of plasma cortisol in rainbow trout (*Oncorhynchus mykiss*). *J. Exp. Biol.* 205 3679–3687. 12409494 10.1242/jeb.205.23.3679

[B36] Lepage O. Vilchez I. M. Pottinger T. G. Winberg S. (2003). Time-course of the effect of dietary L-tryptophan on plasma cortisol levels in rainbow trout *Oncorhynchus mykiss*. *J. Exp. Biol.* 206 3589–3599. 10.1242/Jeb.00614 12966050

[B37] Li P. Mai K. S. Trushenski J. Wu G. Y. (2009). New developments in fish amino acid nutrition: towards functional and environmentally oriented aquafeeds. *Amino Acids* 37 43–53. 10.1007/s00726-008-0171-1 18751871

[B38] Livak K. J. Schmittgen T. D. (2001). Analysis of relative gene expression data using real-time quantitative PCR and the 2-ΔΔCT Method. *Methods* 25 402–408. 10.1006/meth.2001.1262 11846609

[B39] Machado M. Azeredo R. Diaz-Rosales P. Afonso A. Peres H. Oliva-Teles A. (2015). Dietary tryptophan and methionine as modulators of European seabass (*Dicentrarchus labrax*) immune status and inflammatory response. *Fish Shellfish Immun.* 42 353–362. 10.1016/j.fsi.2014.11.024 25463296

[B40] Manning T. S. Gibson G. R. (2004). Prebiotics. *Best Pract. Res. Clin. Gastroenterol.* 18 287–298. 10.1053/ybega.2004.445 15123070

[B41] Martos-Sitcha J. A. Wunderink Y. S. Straatjes J. Skrzynska A. K. Mancera J. M. Martinez-Rodriguez G. (2014). Different stressors induce differential responses of the CRH-stress system in the gilthead sea bream (*Sparus aurata*). *Comp. Biochem. Phys. A* 177 49–61. 10.1016/j.cbpa.2014.07.021 25088183

[B42] Merino G. E. Piedrahita R. H. Conklin D. E. (2007). The effect of fish stocking density on the growth of California halibut (*Paralichthys californicus*) juveniles. *Aquaculture* 265 176–186. 10.1016/j.aquaculture.2007.01.028

[B43] Moffett J. R. Namboodiri M. A. (2003). Tryptophan and the immune response. *Immunol. Cell Biol.* 81 247–265. 10.1046/j.1440-1711.2003.t01-1-01177.x 12848846

[B44] Moltesen M. Laursen D. C. Thornqvist P. O. Andersson M. A. Winberg S. Hoglund E. (2016). Effects of acute and chronic stress on telencephalic neurochemistry and gene expression in rainbow trout (*Oncorhynchus mykiss*). *J. Exp. Biol.* 219 3907–3914. 10.1242/jeb.139857 27802140

[B45] Mommsen T. P. Vijayan M. M. Moon T. W. (1999). Cortisol in teleosts: dynamics, mechanisms of action, and metabolic regulation. *Rev. Fish Biol. Fisher* 9 211–268. 10.1023/A:1008924418720

[B46] Munn D. H. Mellor A. L. (2013). Indoleamine 2,3 dioxygenase and metabolic control of immune responses. *Trends Immunol.* 34 137–143. 10.1016/J.It.2012.10.001 23103127 PMC3594632

[B47] Navarro S. Soletto L. Puchol S. Rotllant J. Soengas J. L. Cerda-Reverter J. M. (2016). 60 YEARS OF POMC POMC: an evolutionary perspective. *J. Mol. Endocrinol.* 56 T113–T118. 10.1530/Jme-15-0288 26671895

[B48] Nayak S. K. (2010). Probiotics and immunity: a fish perspective. *Fish Shellfish Immun.* 29 2–14. 10.1016/j.fsi.2010.02.017 20219683

[B49] Nonaka M. Smith S. L. (2000). Complement system of bony and cartilaginous fish. *Fish Shellfish Immun.* 10 215–228. 10.1006/fsim.1999.0252 10938735

[B50] Pérez-Maceira J. J. Otero-Rodiño C. Mancebo M. J. Soengas J. L. Aldegunde M. (2016). Food intake inhibition in rainbow trout induced by activation of serotonin 5-HT2C receptors is associated with increases in POMC, CART and CRF mRNA abundance in hypothalamus. *J. Comp. Physiol. B* 186 313–321. 10.1007/s00360-016-0961-9 26832922

[B51] Philip A. M. Kim S. D. Vijayan M. M. (2012). Cortisol modulates the expression of cytokines and suppressors of cytokine signaling (SOCS) in rainbow trout hepatocytes. *Dev. Comp. Immunol.* 38 360–367. 10.1016/j.dci.2012.07.005 22878426

[B52] Quade M. J. Roth J. A. (1997). A rapid, direct assay to measure degranulation of bovine neutrophil primary granules. *Vet. Immunol. Immunopathol.* 58 239–248. 10.1016/S0165-2427(97)00048-2 9436268

[B53] Rafatnezhad S. Falahatkar B. Gilani M. H. T. (2008). Effects of stocking density on haematological parameters, growth and fin erosion of great sturgeon (*Huso huso*) juveniles. *Aquac. Res.* 39 1506–1513. 10.1111/j.1365-2109.2008.02020.x

[B54] Robb T. Abrahams M. V. (2003). Variation in tolerance to hypoxia in a predator and prey species: an ecological advantage of being small? *J. Fish Biol.* 62 1067–1081. 10.1046/j.1095-8649.2003.00097.x

[B55] Saeij J. P. J. Verburg-van Kemenade L. B. M. van Muiswinkel W. B. Wiegertjes G. F. (2003). Daily handling stress reduces resistance of carp to *Trypanoplasma borreli*: in vitro modulatory effects of cortisol on leukocyte function and apoptosis. *Dev. Comp. Immunol.* 27 233–245. 10.1016/S0145-305x(02)00093-9 12590974

[B56] Salas-Leiton E. Anguis V. Manchado M. Canavate J. P. (2008). Growth, feeding and oxygen consumption of Senegalese sole (*Solea senegalensis*) juveniles stocked at different densities. *Aquaculture* 285 84–89. 10.1016/j.aquaculture.2008.08.001

[B57] Salas-Leiton E. Anguis V. Martin-Antonio B. Crespo D. Planas J. V. Infante C. (2010). Effects of stocking density and feed ration on growth and gene expression in the Senegalese sole (*Solea senegalensis*): potential effects on the immune response. *Fish Shellfish Immun.* 28 296–302. 10.1016/j.fsi.2009.11.006 19909816

[B58] Schram E. Van der Heul J. W. Kamstra A. Verdegem M. C. J. (2006). Stocking density-dependent growth of Dover sole (*Solea solea*). *Aquaculture* 252 339–347. 10.1016/j.aquaculture.2005.07.011

[B59] Seasholtz A. F. Valverde R. A. Denver R. J. (2002). Corticotropin-releasing hormone-binding protein: biochemistry and function from fishes to mammals. *J. Endocrinol.* 175 89–97. 10.1677/joe.0.1750089 12379493

[B60] Silva J. M. G. Espe M. Conceição L. E. C. Dias J. Costas B. Valente L. M. P. (2010). Feed intake and growth performance of Senegalese sole (*Solea senegalensis* Kaup, 1858) fed diets with partial replacement of fish meal with plant proteins. *Aquacult. Res.* 41 e20–e30. 10.1111/j.1365-2109.2009.02451.x

[B61] Spinedi E. Negrovilar A. (1983). Serotonin and Acth release - direct effects at the anterior-pituitary level and potentiation of Avp-induced Acth release. *Fed Proc.* 42 459–459. 10.1210/endo-112-4-1217 6299688

[B62] Sunyer J. O. Tort L. (1995). Natural hemolytic and bactericidal activities of sea bream *Sparus aurata* serum are effected by the alternative complement pathway. *Vet. Immunol. Immunopathol.* 45 333–345. 10.1016/0165-2427(94)05430-Z 7676614

[B63] Takahashi A. Kobayashi Y. Mizusawa K. (2013). The pituitary-interrenal axis of fish: a review focusing on the lamprey and flounder. *Gen. Comp. Endocrinol.* 188 54–59. 10.1016/j.ygcen.2013.03.005 23524003

[B64] Tort L. (2011). Stress and immune modulation in fish. *Dev. Comp. Immunol.* 35 1366–1375. 10.1016/j.dci.2011.07.002 21782845

[B65] Varsamos S. Flik G. Pepin J. F. Bonga S. E. W. Breuil G. (2006). Husbandry stress during early life stages affects the stress response and health status of juvenile sea bass, *Dicentrarchus labrax*. *Fish Shellfish Immun.* 20 83–96. 10.1016/j.fsi.2005.04.005 15961320

[B66] Verburg-Van Kemenade B. M. L. Ribeiro C. M. S. Chadzinska M. (2011). Neuroendocrine-immune interaction in fish: differential regulation of phagocyte activity by neuroendocrine factors. *Gen. Comp. Endocrinol.* 172 31–38. 10.1016/j.ygcen.2011.01.004 21262228

[B67] Winberg S. Lepage O. (1998). Elevation of brain 5-HT activity, POMC expression, and plasma cortisol in socially subordinate rainbow trout. *Am. J. Physiol.* 274 R645–R654. 10.1152/ajpregu.1998.274.3.R645 9530229

[B68] Winberg S. Nilsson A. Hylland P. Söderstöm V. Nilsson G. E. (1997). Serotonin as a regulator of hypothalamic-pituitary-interrenal activity in teleost fish. *Neurosci. Lett.* 230 113–116. 10.1016/S0304-3940(97)00488-6 9259477

[B69] Winberg S. Overli O. Lepage O. (2001). Suppression of aggression in rainbow trout (*Oncorhynchus mykiss*) by dietary L-tryptophan. *J. Exp. Biol.* 204 3867–3876. 11807104 10.1242/jeb.204.22.3867

[B70] Wunderink Y. S. de Vrieze E. Metz J. R. Halm S. Martinez-Rodriguez G. Flik G. (2012). Subfunctionalization of POMC paralogues in Senegalese sole (*Solea senegalensis*). *Gen. Comp. Endocrinol.* 175 407–415. 10.1016/j.ygcen.2011.11.026 22142534

[B71] Wunderink Y. S. Engels S. Halm S. Yufera M. Martinez-Rodriguez G. Flik G. (2011). Chronic and acute stress responses in Senegalese sole (*Solea senegalensis*): the involvement of cortisol, CRH and CRH-BP. *Gen. Comp. Endocrinol.* 171 203–210. 10.1016/j.ygcen.2011.01.010 21291885

[B72] Yarahmadi P. Miandare H. K. Hoseinifar S. H. Gheysvandi N. Akbarzadeh A. (2015). The effects of stocking density on hemato-immunological and serum biochemical parameters of rainbow trout (*Oncorhynchus mykiss*). *Aquacult. Int.* 23 55–63. 10.1007/s10499-014-9797-z

